# Impact of Marginal Misfit in Implant‐Supported Fixed Dental Prostheses on Peri‐Implant Bone Levels: A Retrospective Quantitative Analysis

**DOI:** 10.1111/clr.70053

**Published:** 2025-10-04

**Authors:** Emilio Couso‐Queiruga, Christoph A. Ramseier, Vivianne Chappuis, Simone F. M. Janner M, Daniel Buser, Urs Brägger, Giovanni E. Salvi

**Affiliations:** ^1^ Department of Oral Surgery and Stomatology University of Bern School of Dental Medicine Bern Switzerland; ^2^ Department of Periodontology University of Bern School of Dental Medicine Bern Switzerland; ^3^ Department of Reconstructive Dentistry and Gerodontology School of Dental Medicine Bern Switzerland

**Keywords:** bone resorption, dental digital radiography, dental implants, implant shoulder bone distance, implant‐supported prostheses, outcome assessment

## Abstract

**Objectives:**

To evaluate the impact of the marginal fit of implant‐supported prostheses (ISP) on peri‐implant bone levels. Additionally, the study aimed to determine a clinically relevant threshold for the radiographic vertical misfit gap at the ISP, when present, and to identify potential risk factors associated with changes in bone levels.

**Methods:**

This study involved subjects who received ISPs for tooth replacement therapy. Standardized intraoral periapical radiographs were taken 10 years after loading to assess the radiographic distance between the implant shoulder and the most coronal point of crestal bone (DIB). ISP marginal gaps were categorized as no gap or gap, with vertical dimensions categorized as 0 mm, > 0‐ < 0.1 mm, and ≥ 0.1 mm. A multivariable linear mixed‐effect model was applied to control for potential confounders.

**Results:**

A total of 301 patients and 505 implants with a 10.6 ± 0.7 years follow‐up were analyzed. ISPs without gaps exhibited statistically significantly lower DIB values (3.22 ± 0.8 mm) than those with gaps (3.43 ± 0.6 mm; *p* = 0.001). Gaps ≥ 0.1 mm were associated with statistically significantly higher DIB values (3.45 ± 0.7 mm; *p* = 0.001) compared with gaps between > 0 and < 0.1 mm (3.36 ± 0.5 mm; *p* = 0.001), or no gaps (3.22 ± 0.7 mm; *p* = 0.001). Each increment of 0.1 mm in the vertical crestal gap corresponded to a significant increase in DIB values (0.08 mm; *p* = 0.03). Finally, smoking and a history of periodontitis were independent risk factors for increased DIB.

**Conclusions:**

Marginal misfit of ISP affects peri‐implant bone stability, with gaps ≥ 0.1 mm linked to higher DIB. Smoking and periodontitis are independent risk factors for increased DIB.

## 1 Introduction

Implant‐supported prostheses (ISP) are highly effective in replacing missing teeth, restoring oral function, health, esthetics, and comfort, ultimately improving patients' quality of life (Braun et al. 2025; Buser et al. 2012; Duong et al. 2022; Howe et al. 2019; Raabe et al. 2024). However, the high prevalence of peri‐implant diseases and the unpredictability of resolving the condition through non‐surgical or surgical therapies remain critical challenges (Apaza‐Bedoya et al. 2024; Couso‐Queiruga et al. 2024; Diaz et al. 2022; Garaicoa‐Pazmino et al. 2025; Romandini et al. 2021).

Diagnosing peri‐implant diseases has traditionally relied on clinical parameters (e.g., probing depths (PD), presence or absence of bleeding on probing) and radiographic assessment of crestal bone levels (Lang and Berglundh 2011; Mombelli and Lang 1998; Monje and Salvi 2024). However, inconsistencies in the definitions of case criteria and challenges in ensuring external validity have hindered the standardization of diagnosis (Figuero et al. 2014; Garaicoa‐Pazmino et al. 2025). The 2017 World Workshop on the Classification of Periodontal and Peri‐implant Diseases and Conditions established consensus‐based case definitions and diagnostic guidelines to address these issues (Renvert et al. 2018). Therefore, in cases of peri‐implant inflammation, a definitive diagnosis should be based on the extent and severity of radiographic crestal bone loss (Herrera et al. 2023; Monje and Salvi 2024; Renvert et al. 2018).

Multiple factors influence the presence of radiographic bone loss beyond physiological remodeling. Studies have identified associations with early interproximal thread exposure (Ravidà et al. 2023; Windael et al. 2021), host‐immune responses such as diabetes (Chrcanovic et al. 2014; French et al. 2019), heavy smoking (French et al. 2019), implant‐abutment connection types (Lemos et al. 2018), abutment height (Del Amo et al. 2024; Galindo‐Moreno et al. 2014), history of periodontal disease (Trombelli et al. 2024), inadequate oral hygiene, and lack of supportive peri‐implant care (Carra et al. 2023), among others. Additionally, iatrogenic‐related factors, including excess cement, over‐contoured ISP, implant malposition, and inadequate restoration abutment‐seating (Lang and Berglundh 2011), have also been associated with radiographic bone loss.

Despite these findings, the role of other prosthetic‐related factors in peri‐implant bone levels remains insufficiently explored (Mattheos et al. 2021). Therefore, the primary outcome of this study was to evaluate the impact of marginal misfit of ISP on radiographic crestal bone levels. The secondary objective was to establish a clinically relevant threshold for the radiographic vertical misfit gap at the ISP and to identify potential risk factors associated with radiographic bone loss.

## 2 Materials and Methods

### 2.1 Experimental Design, Study Center, Ethical Approval, and Registration

The study was designed as a retrospective study. The clinical examinations were conducted in the Departments of Periodontology, Oral Surgery and Stomatology, and Reconstructive Dentistry and Gerodontology, School of Dental Medicine, University of Bern, Switzerland, between May 1997 and January 2001. The study complied with the Reporting of Observational Studies in Epidemiology (STROBE) guidelines (von Elm et al. 2008) and was conducted according to the principles of the Helsinki Declaration 1975, as revised in 2013 (“World Medical Association Declaration of Helsinki: ethical principles for medical research involving human subjects,” 2013). The protocol was submitted to and approved by the standing Ethics Committee for Clinical Studies of the State of Bern, Switzerland (KEK‐BE‐No. 2025‐00209).

### 2.2 Eligibility Criteria and Recruitment

The health records of adult subjects who underwent tooth replacement therapy with dental implants and ISPs between May 1997 and January 2001 were reviewed for potential inclusion in this study (Buser et al. 2012). The inclusion criteria were: (1) age ≥ 18 years; (2) partial edentulism; (3) placement of a soft‐tissue level implant to replace at least one missing tooth; (4) restoration with a cement or screw‐retained ISP; and (5) availability of high‐quality periapical radiographs taken at least 10 years post‐loading. Exclusion criteria consisted of (1) inadequate quality of periapical radiographic images and (2) patients who denied their clinical and radiographic data from being used for further analysis.

Patients who expressed interest in participating in the study were initially pre‐screened by the clinical coordinator. During the subsequent in‐person clinical screening, candidates were provided detailed information about the study's purpose and timeline. All participants were required to read and understand the informed consent form, which thoroughly outlined the study design, potential benefits, and possible risks associated with participation before signing it.

### 2.3 Clinical Procedures and Digital Data Acquisition and Collection

All clinical procedures were performed in the Departments of Periodontology, Oral Surgery and Stomatology, and Reconstructive Dentistry and Gerodontology, as previously described in a study involving this patient cohort (Buser et al. 2012). In summary, tissue‐level implants were placed in a prosthetically driven position, achieving adequate primary stability. The polished neck surface of the implants was positioned slightly subcrestally.

At the 10‐year follow‐up, information related to the presence or absence of suppuration, modified Plaque Index (mPLI) (Mombelli et al. 1987), modified Sulcus Bleeding Index (mSBI) (Mombelli et al. 1987), PD at six sites per implant, and apical migration of the mucosal margin was obtained from the patient's dental history (Buser et al. 2012). Finally, patient demographics, personal health records, history of periodontitis, participation in a supportive peri‐implant care program, implant characteristics and location, and data on surgical‐related variables were also collected through a structured questionnaire.

A standardized periapical radiograph, centered on the implant of interest, was obtained at least 10 years post‐loading for assessing the distance between the implant shoulder and the highest point of the crestal bone (DIB) and for diagnostic evaluation. To ensure high‐quality data, two independent and calibrated examiners (V.C. and S.F.M.J.) evaluated the radiographs using open‐source software (ImageJ, U.S. NIH, Bethesda, MD, USA) in a darkened room. The calibration session was performed with the first 50 consecutive images, analyzed twice in 4 weeks. In cases where individual measurements differed by more than 0.3 mm, the two authors verified final data accuracy and consistency by reaching a consensus on the exact position of the distance to the implant shoulder and repeating the measurements to ensure precision. For each radiograph, a horizontal line was drawn at the implant shoulder. Vertical lines were then drawn from the most coronal bone level to determine the mesial and distal DIB, ensuring the lines were perpendicular to the horizontal line (Saito et al. 2021). Finally, the total DIB was obtained for each implant as the mean of the mesial and distal vertical DIB. The known thread pitch distances of each implant were used to convert pixel measurements into millimeters. All measurement lines were carefully aligned parallel to the implant's long axis to minimize error during assessment.

**FIGURE 1:**
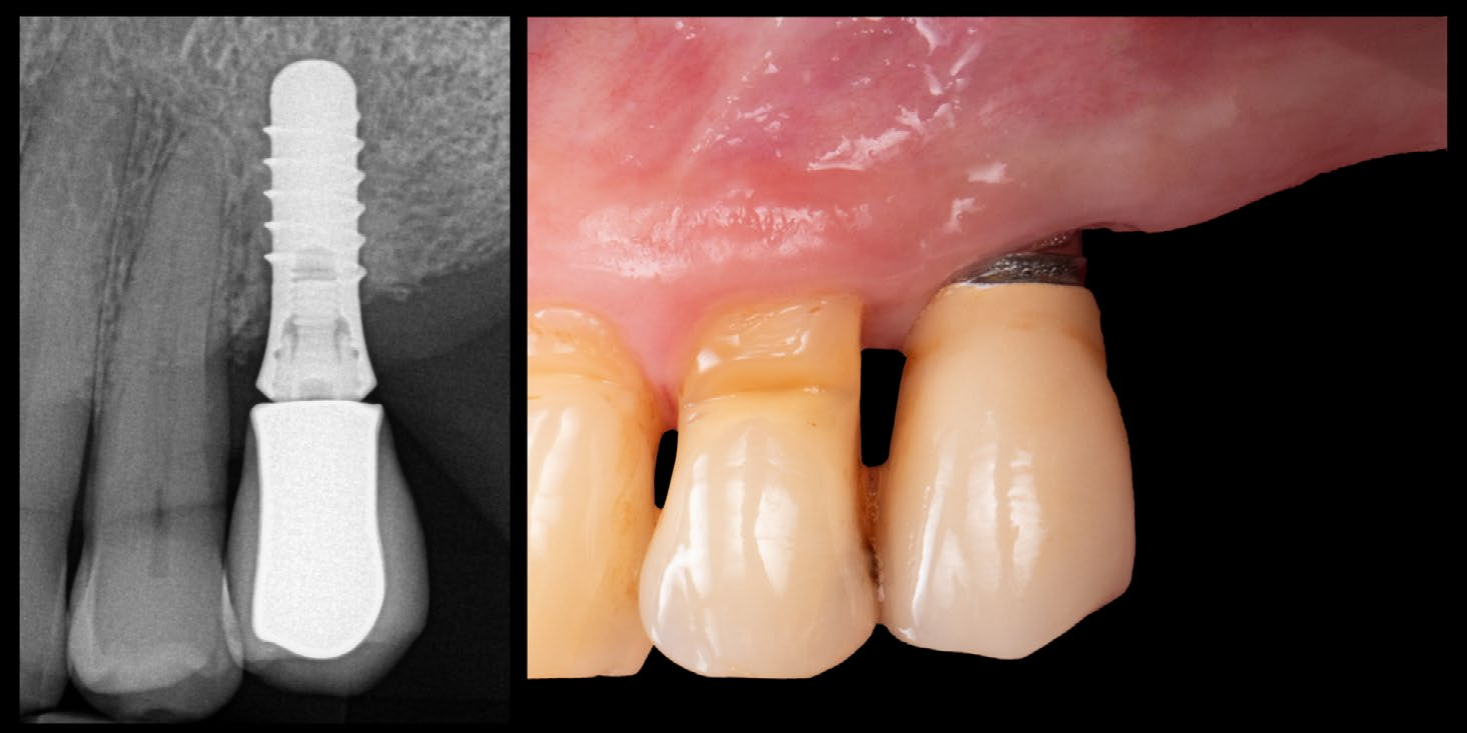
A clinical case illustrating a radiographic misfit of the implant‐supported prosthesis and peri‐implant marginal mucosal dehiscence, exposing the implant surface.

Additionally, the presence or absence of prosthetic gap categories (GapCAT) at the ISP was radiographically assessed and categorized as either “no gap” or “presence of gap”. Vertical dimensions (GapDIM) were categorized as 0 mm, > 0–< 0.1 mm, and ≥ 0.1 mm. GapDIM on ISP presenting a prosthetic misfit was measured in mm. Similarly, the presence or absence of excess cementum was noted as a dichotomous variable. An example of a clinical and radiographic misfit is presented in Figure 1.

### 2.4 Statistical Analysis

Descriptive statistics were used to summarize the baseline characteristics of the study population. Continuous variables were expressed as means and standard deviations, while categorical variables were reported as frequencies and percentages. Group comparisons of mean DIB between implants with and without ISP gaps, and between different gap dimensions, were performed using the following inferential statistical tests: For normally distributed data, independent *t*‐tests were used to compare the mean DIB between implants with and without ISP gaps. For comparing multiple gap dimensions, one‐way ANOVA was applied. For non‐normally distributed data, Mann–Whitney *U* tests were used for pairwise comparisons between two groups (e.g., ISP with vs. ISP without gaps), and Kruskal‐Wallis tests were used to compare the DIB across multiple gap dimensions. The assumption of normality for continuous variables was assessed using the Shapiro–Wilk test. For non‐normally distributed data, appropriate non‐parametric tests were applied. A multivariable linear mixed‐effects model was applied to determine the relationship between various predictor variables and the dependent variable mean DIB. The model accounted for repeated measurements within individuals by including patient‐specific random intercepts. The fixed effects of the predictor variables were evaluated for statistical significance using Wald tests. Statistical significance was defined as *p* < 0.05, and estimates, standard errors, 95% confidence intervals, and *p*‐values were reported for each fixed effect. All analyses were conducted using RStudio: Integrated Development Environment for R (RStudio, PBC, Boston, MA 2024.09.0).

### 2.5 Sample Size Calculation

Data from a previous investigation reporting this cohort of patients was used to predict the total number of implants that could be included in this study (Buser et al. 2012). Considering an anticipated total of 486 implants in the present study, accounting for a 5% dropout rate, a power analysis was performed to ensure adequate statistical power. Initially, to detect a 0.2 mm gap between ISP with and without GapCAT, a mixed linear regression model was used. Given the small‐to‐moderate effect size (Cohen's *d* = 0.33), a significance level of 0.05, and 95% power, the sample size calculation indicated that at least 125 implants per group, 250 in total, would be required to achieve statistical significance. This sample size was further adjusted for random effects, accounting for subject‐level variability and potential repeated measures.

## 3 Results

### 3.1 Population and Sample Characteristics

The outcomes of 303 patients with 511 implants were initially screened. However, the radiographs of two patients with six implants could not be accurately assessed in terms of the prosthetic fit of the ISP, so they were excluded from the analysis. Consequently, the final study cohort comprised 301 patients with 505 implants. The cohort consisted of 142 (47.2%) males and 159 females (52.8%) with a mean age of 50.6 ± 15.1 years. A total of 439 (87.8%) participants were non‐smokers, while 61 (12.2%) were smokers, with a mean smoking history of 18.5 ± 11.0 pack‐years. Furthermore, 25 (5.0%) patients were diagnosed with diabetes mellitus, 17 (3.4%) reported osteoporosis, and 326 (68.6%) reported consuming alcohol. The mean intervals for supportive peri‐implant care per year were 1.74 ± 0.90 visits, and 103 (35.6%) patients had a history of periodontitis. A summary of baseline population characteristics is presented in Table 1.

**TABLE 1:** Summary of baseline population characteristics.

Population characteristics	Mean (SD)
Age (years)	50.6 (15.1)
Gender	
Female	142 (47.2%)
Male	159 (52.8%)
Smokers	61 (12.2%)
Diabetes mellitus	25 (5.0%)
Osteoporosis	17 (3.4%)
Alcohol consumption	326 (68.6%)
Supportive peri‐implant care per year	1.74 (0.90)
History of periodontitis	103 (35.6%)

*Note:* Data presented as mean with its standard deviation (SD) for continuous, and *n* (%) for count data.

The mean follow‐up of the implants was 10.6 ± 0.7 years. Implant diameters were as follows: 3.3 mm (*n* = 16), 4.1 mm (*n* = 277), and 4.8 mm (*n* = 212). Implant platform dimensions were 3.5 mm (*n* = 16), 4.8 mm (*n* = 418), and 6.5 mm (*n* = 71). Implant lengths were as follows: 6 mm (*n* = 13), 8 mm (*n* = 81), 10 mm (*n* = 236), 12 mm (*n* = 173), and 14 mm (*n* = 2). Implant neck heights were 2.8 mm (*n* = 432) and 1.8 mm (*n* = 73).

**FIGURE 2:**
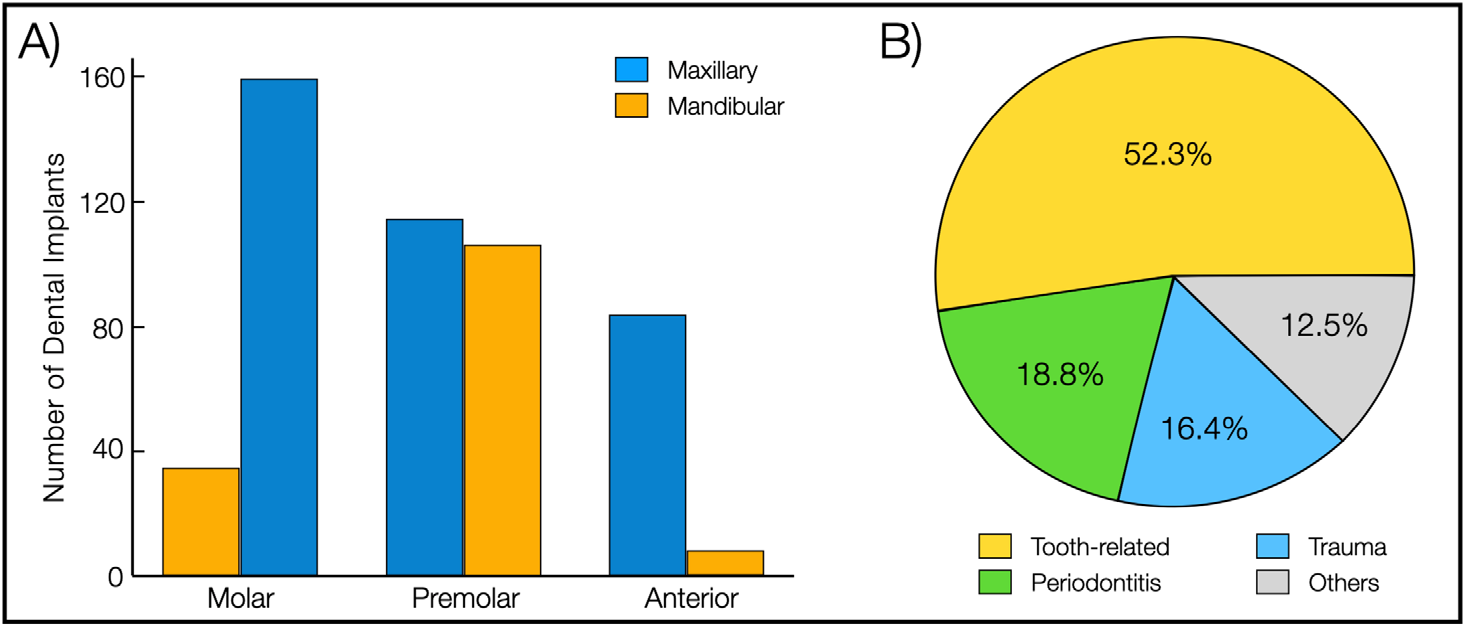
Data representation of implant distribution and causes of tooth loss. (A) Bar chart showing the number of implants placed across various locations. (B) Pie chart displaying the etiological causes for tooth loss in the study group.

In maxillary areas, implants were placed in the molar (*n* = 35), premolar (*n* = 114), and anterior (*n* = 83) areas. Similarly, in the mandible, implants were located in the molar (*n* = 159), premolar (*n* = 106), and anterior (*n* = 8) areas. The most common reasons for tooth loss (*n* = 226) were tooth‐related complications, including endodontic complications, carious lesions, and fractures, followed by periodontitis (*n* = 81), trauma (*n* = 71), and others (*n* = 54). The etiology of tooth loss of the remaining missing teeth was unknown (*n* = 86). Trauma was the primary cause of anterior tooth loss. A distribution of implant placement locations and tooth loss etiology is presented in Figure 2.

A total of 355 implants were placed without additional bone augmentation. However, 90 implants required staged (*n* = 37) or simultaneous horizontal bone augmentation (*n* = 53). Additionally, 68 sites underwent sinus floor elevation, either through a simultaneous lateral (*n* = 8), crestal (*n* = 23), or staged (*n* = 37) approach.

The mean mPLI and mSBI values were 0.6 ± 0.6 and 1.3 ± 0.6, respectively. Only two implants showed suppuration on probing. The mean PD values were 3.3 ± 1.1 mm, while the mean apical migration of the mucosal margin was 0.4 ± 1.3 mm. Sample characteristics are displayed in Table 2.

**TABLE 2:** Summary of sample characteristics.

Sample characteristics	Mean (SD)
Time in function (years)	10.6 (0.7)
Implant diameters	3.3 mm (*n* = 16), 4.1 mm (*n* = 277), 4.8 mm (*n* = 212)
Implant platform dimensions	3.5 mm (*n* = 16), 4.8 mm (*n* = 418), 6.8 mm (*n* = 71)
Implant lengths	6 mm (*n* = 13), 8 mm (*n* = 81), 10 mm (*n* = 236), 12 mm (*n* = 173), 14 mm (*n* = 2)
Implant neck heights	2.8 mm (*n* = 432), 1.8 mm (*n* = 73)
Location	
Maxilla	Molar (*n* = 35), premolar (*n* = 113), anterior (*n* = 83)
Mandible	Molar (*n* = 159), premolar (*n* = 106), anterior (*n* = 8)
Implants without bone augmentation	355
Bone augmentation procedures	158 (staged (*n* = 37), and simultaneous (*n* = 53), horizontal augmentation, simultaneous lateral (*n* = 8), crestal (*n* = 23), and staged (*n* = 37) sinus floor elevation)
mPLI	0.6 (0.6)
mSBI	1.3 (0.6)
PD (mm)	3.3 (1.1)
Apical migration of mucosal margin (mm)	−0.4 (1.3)

*Note:* Continuous variables are presented as mean ± standard deviation (SD), while categorical variables are expressed as *n* (%).

Abbreviations: Mm, millimeters; mPLI, modified Plaque Index; mSBI, modified Sulcus Bleeding Index; PD, Probing depth. [Correction added on 8 October 2025, after first online publication: The citations of Karoussis et al. in the Table body have been updated to (mm).]

### 3.2 Radiographic Outcomes

**FIGURE 3:**
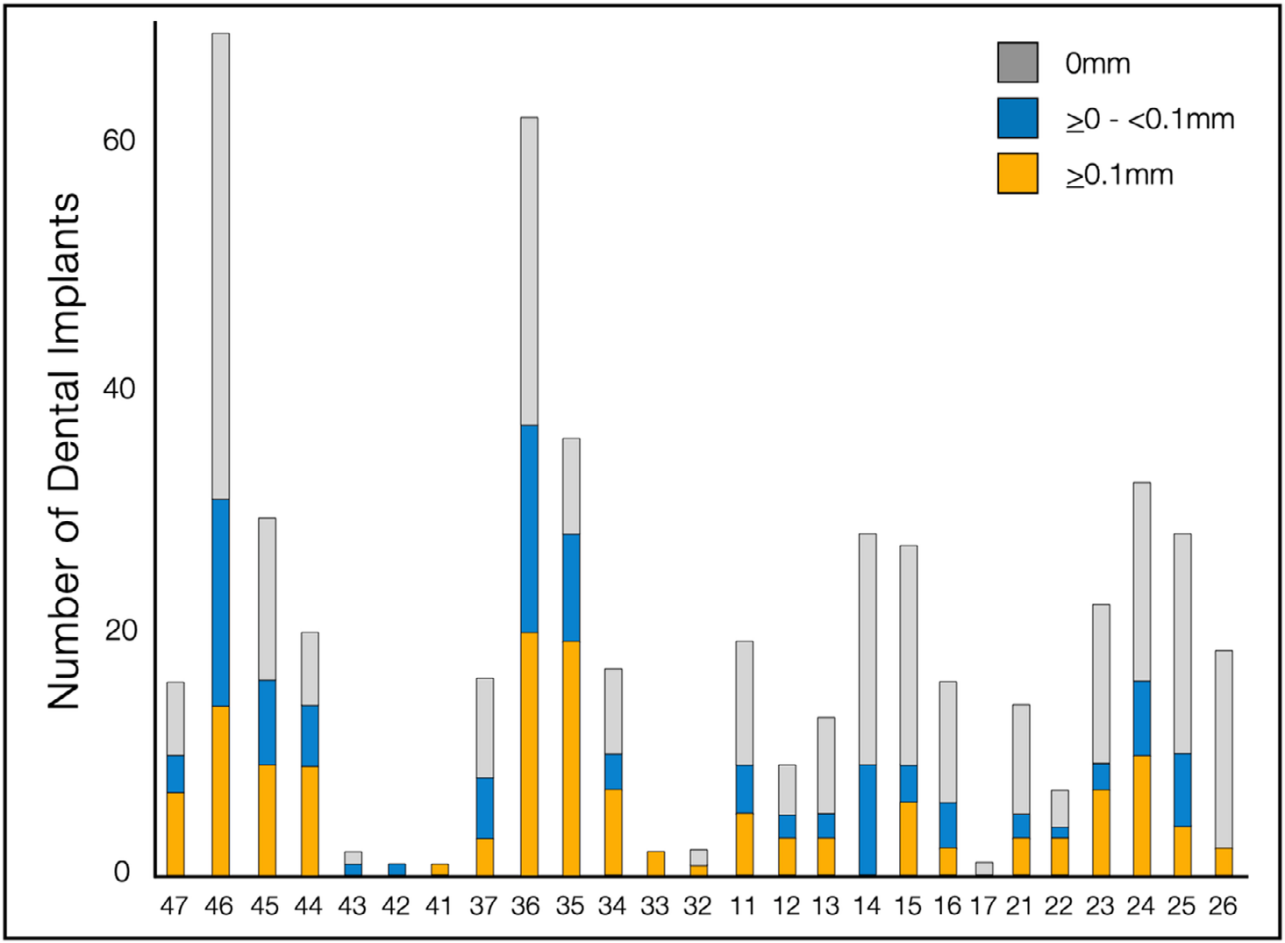
Graphical analysis of gap distribution in relation to the number and locations of dental implants.

The mean DIB values were 3.3 ± 0.7 mm. GapCAT was present in 249 implants (49.3%), of which 159 had a GapDIM mean value > 0.1 mm in the mesial and/or distal sites. The mean GapDIM values at the mesial, distal, and total sites were 0.1 ± 0.1 mm, 0.1 ± 0.2 mm, and 0.1 ± 0.1 mm, respectively, ranging from 0.02 mm to 3.7 mm. ISPs with no gaps exhibited statistically significantly lower DIB values (3.22 ± 0.8 mm) compared to those with gaps (3.43 ± 0.6 mm; *p* = 0.0018). Gaps ≥ 0.1 mm were associated with significantly greater DIB values (3.45 ± 0.7 mm; *p* = 0.001) compared with gaps between > 0 and < 0.1 mm (3.36 ± 0.5 mm; *p* = 0.001), or no gaps (3.22 ± 0.7 mm; *p* = 0.001). The overall distribution of gap and implant locations is depicted in Figure 3. Additionally, larger vertical gap dimensions were associated with increased DIB, with each 0.1 mm increase in gap size corresponding to an average DIB rise of 0.08 mm (*p* = 0.03). Finally, radiographic residual cement was observed in 11 implants. Radiographic outcomes are shown in Table 3.

**TABLE 3:** Summary of radiographic outcomes.

Radiographic outcomes	Mean (SD)
DIB total values (mm)	3.3 (0.7)
GapCAT present	249 implants (49.3%)
Implants with GapDIM > 0.1 mm	159
GapDIM total values (mm)	0.1 (0.1)
DIB values ISP with gaps (mm)	3.43 (0.6)
DIB values ISP without gaps (mm)	3.22 (0.8)
Apical migration of mucosal margin (mm)	−0.4 (1.3)

*Note:* Continuous variables are presented as mean ± standard deviation (SD), while categorical variables are expressed as *n* (%).

Abbreviations: DBI, radiographic distance between the implant shoulder and the most coronal part of the crestal bone; GapCAT, gap category; GapDIM, gap dimensions; ISP, implant‐supported prostheses; Mm, millimeters. [Correction added on 8 October 2025, after first online publication: The citations of Karoussis et al. in the Table body have been updated to (mm).]

### 3.3 Relationship Between DIB and Crestal Bone Levels or Other Variables

A multivariable linear mixed effects model was applied to assess the relationship between various predictor variables and the dependent variable (DIB) as reported in Table 4. Patient‐specific random intercepts were included to account for repeated measurements within individuals.

**TABLE 4:** Results of the linear mixed‐effects model estimate the relationship between predictor variables and crestal bone levels, including fixed and random effects with corresponding statistical estimates.

Variable	Estimate	Standard error	*t*‐Statistic	df	OR	95% CI (Lower)	95% CI (Upper)	*p*
Intercept	2.585	0.697	3.709	412.8	13.261	1.219	3.951	0.0002***
Implant neck height	0.024	0.111	0.215	465.2	1.024	−0.194	0.242	0.8298
GapDIM	0.041	0.038	1.078	458.2	1.042	−0.034	0.115	0.2816
Time in function	0.000	0.004	−0.059	384.2	1.000	−0.009	0.009	0.9526
Implant length	0.035	0.021	1.691	456.4	1.036	−0.006	0.076	0.0916
Implant with simultaneous horizontal augmentation (Yes vs. No) (Ref: No)	0.134	0.125	1.070	452.7	1.144	−0.112	0.380	0.2852
Implant placement with sinus lifting (lateral approach) (Yes vs. No) (Ref: No)	−0.047	0.264	−0.178	467.7	0.954	−0.564	0.470	0.8588
Implant placement with sinus lifting (transcrestal approach) (Yes vs. No) (Ref: No)	−0.538	0.150	−3.583	455.1	0.584	−0.832	−0.244	0.0004***
Implant after staged horizontal augmentation (Yes vs. No) (Ref: No)	0.242	0.129	1.872	451.0	1.274	−0.011	0.496	0.0618
Implant after staged sinus lifting (lateral approach) (Yes vs. No) (Ref: No)	−0.208	0.155	−1.345	454.1	0.812	−0.511	0.095	0.1793
Use of bone grafting material (Yes vs. No) (Ref: No)	0.238	0.318	−0.159	463.6	1.386	−0.385	0.860	0.1540
Diabetes mellitus (Yes vs. No) (Ref: No)	−0.093	0.210	−0.443	198.8	0.911	−0.504	0.318	0.6582
Osteoporosis (Yes vs. No) (Ref: No)	0.410	0.225	1.819	268.9	1.507	−0.032	0.852	0.0700
Smoking (Yes vs. No) (Ref: No)	0.358	0.114	3.128	296.8	1.430	0.134	0.582	0.0019**
Presence of radiographic residual cement (Yes vs. No) (Ref: No)	0.206	0.199	1.039	443.5	1.229	−0.183	0.595	0.2994
History of periodontitis (Yes vs. No) (Ref: No)	0.164	0.049	3.322	297.5	1.178	0.067	0.260	0.0010**
PD	−0.030	0.039	−0.756	457.3	0.971	−0.106	0.047	0.4498
mPLI	−0.115	0.072	−1.594	440.2	0.892	−0.256	0.026	0.1117
mSBI	0.065	0.096	0.680	456.2	1.067	−0.122	0.253	0.4967

Abbreviations: CI, confidence interval; Df, degrees of freedom; GapDIM, gap dimensions; mPLI, modified plaque index; mSBI, modified Sulcus Bleeding Index; OR, odds ratio; PD, probing depth; Ref, reference category.

* *p* < 0.05.

** *p* < 0.01.

*** *p* < 0.001.

The model intercept was significant (estimate = 2.6 ± 0.7 mm, *p* < 0.001), indicating the mean DIB total mean values for the reference group were 2.6 ± 0.7 mm. Among the predictor variables, smoking (estimate = 0.36 ± 0.1 mm, *p* = 0.001) and history of periodontitis (estimate = 0.16 ± 0.1 mm, *p* = 0.001) were significantly associated with increased DIB total mean values, suggesting that these factors contributed to greater bone loss. Conversely, the surgical technique, specifically, the sinus floor elevation procedure via a crestal approach, was significantly associated with a decrease in DIB mean (estimate = −0.54 ± 0.15 mm, *p* < 0.001), indicating a potential protective effect on radiographic crestal bone levels.

Other variables, including the neck height (i.e., 1.8 and 2.8 mm) (estimate = 0.02 ± 0.1 mm, *p* = 0.83) and GapDIM (estimate = 0.04 ± 0.04 mm, *p* = 0.28), did not show significant associations with DIB total mean values. Similarly, the use of bone grafting material (estimate = 0.24 ± 0.3 mm, *p* = 0.15) and diabetes (estimate = −0.1 ± 0.2 mm, *p* = 0.65) were not significantly correlated with DIB total mean values. The confidence intervals (95% CI) for most non‐significant predictors included zero, reinforcing the lack of strong evidence for their effect on DIB.

## 4 Discussion

This retrospective study aimed to evaluate the effect of the prosthetic fit of ISP on crestal bone levels. Secondary objectives included establishing a clinically relevant threshold for the radiographic misfit gap at the ISP interface and assessing the influence of other potential risk factors on crestal bone levels. To our knowledge, this is the first study to report on the impact of prosthetic misfit on DIB. The main findings indicated that ISPs with a marginal misfit gap exhibited significantly greater DIB compared to those with no gap. Additionally, larger GapDIM were associated with increased DIB. Finally, smoking and a history of periodontitis were identified as independent risk factors contributing to higher DIB values, whereas transcrestal sinus floor elevation served as a protective factor. These findings emphasize the importance of a high‐precision fit to avoid marginal misfit gaps at the implant‐abutment interface and address specific risk factors in daily clinical practice.

Radiographic assessments of DIB revealed that ISPs with misfit gaps exhibited a greater increase in DIB values as compared to sites without gaps. Previous studies suggested that ISP misfit may induce higher stress at the bone‐implant‐prosthetic interface, potentially leading to technical and biological complications (Katsoulis et al. 2017; Saleh et al. 2022). Additionally, these gaps may serve as biofilm‐retentive reservoirs, leading to an inflammatory response in the peri‐implant soft tissues and contributing to radiographic crestal bone loss (Broggini et al. 2006; Hermann et al. 2001; King et al. 2002; Ujiie et al. 2012). The accumulation of bacterial byproducts and endotoxins can further stimulate the expression of proinflammatory cytokines, promoting osteoclast chemotaxis and exacerbating bone resorption at the coronal aspect, thereby increasing the risk of peri‐implant disease development (Insua et al. 2017). Therefore, a precise prosthetic fit and thorough clinical and radiographic follow‐up are essential to ensure optimal outcomes and maintain peri‐implant health.

The analysis of GapDIM in the present study demonstrated a positive correlation between larger GapDIM and increased DIB. Specifically, ISPs with a gap size of ≥ 0.1 mm were associated with significantly greater DIB values compared to ISPs with smaller or no gaps. Notably, an average DIB increase of 0.08 mm was observed after a 10‐year follow‐up period for each 0.1 mm increase in gap size. To the best of the authors' knowledge, this is the first study to estimate a specific threshold linking GapDIM dimensions to DIB values. We used a continuous outcome measure (DIB) and applied a multivariable linear mixed‐effects model instead of categorical thresholds (e.g., DIB > 3.5 mm), as this approach is more clinically intuitive. This approach allowed us to adjust for confounding variables (e.g., smoking and history of periodontitis) while preserving the full range of variability in the outcome measure. Dichotomizing continuous variables can reduce statistical power and obscure dose–response relationships. Our model‐based analysis provides a more nuanced understanding of the relationship between prosthetic misfit and crestal bone levels. Future studies may explore the use of categorical outcomes as a complementary tool for communicating risk to patients. However, given the complexity of influencing factors and the absence of standardized methodologies to assess the impact of misfits on biological complications, these findings should be interpreted with caution (Saleh et al. 2022). This consideration is particularly relevant given that all analyzed implants were of tissue‐level type, where the distance from the ISP misfit to the most coronal part of the alveolar bone crest is generally greater than in bone‐level implants. This could explain why our observations failed to yield an association between neck height and increased DIB values. However, GapDIM and the presence/absence of gaps may have a more pronounced impact on bone‐level implants, depending on apico‐coronal depth position, where the selection of the abutment height and the dimensions of the supracrestal tissue height could play a critical role in the development of crestal bone loss (Borges et al. 2018; de Siqueira et al. 2020; Del Amo et al. 2024; Galindo‐Moreno et al. 2014; Pico et al. 2019; Saleh et al. 2022; Spinato et al. 2019).

The evaluation of potential risk factors for radiographic changes in bone levels identified significant associations between smoking, a history of periodontitis, and increased DIB values. Previous studies indicated the detrimental effects of smoking on crestal bone levels (Bahrami et al. 2017; Galindo‐Moreno et al. 2005; Lindquist et al. 1997). However, the relationship between smoking and peri‐implantitis remains inconclusive, with conflicting reports on its association (Dvorak et al. 2011; Marrone et al. 2013; Rinke et al. 2011; Roos‐Jansåker et al. 2006). Hence, establishing a direct causal link is challenging due to inconsistencies in smoker classification, reliance on self‐reported smoking status, study design limitations, and the lack of well‐controlled longitudinal studies (Carra et al. 2023; Herrera et al. 2023; Schwarz et al. 2018). Similarly, a history of periodontitis was associated with increased DIB values in affected patients. While this finding aligns with previous studies, long‐term adherence to a regular supportive peri‐implant care program appears to mitigate the risk of further biological complications as observed in this study (Karoussis et al. 2003; A. Roccuzzo et al. 2022; M. Roccuzzo et al. 2010; Roos‐Jansåker et al. 2006; Weigel et al. 2023). Nevertheless, robust evidence from longitudinal and cross‐sectional studies indicated that a history of periodontitis is a significant risk factor for the development of radiographic bone loss and peri‐implantitis (Schwarz et al. 2018). Finally, transcrestal sinus floor elevation appears to have a protective effect on crestal bone levels. However, current evidence does not indicate significant clinical and radiographic differences between the transcrestal and other techniques, such as the lateral window approach, as treatment alternatives for the edentulous posterior maxilla (Raghoebar et al. 2019; Shi et al. 2023; Starch‐Jensen and Jensen 2017). Therefore, this observation may be influenced by a spurious correlation, potentially attributable to sample size limitations.

This study has several limitations. First, only tissue‐level implants from a single manufacturer were analyzed, limiting the generalizability of our findings to other implant types and systems. However, the selected sample ensures homogeneity and minimizes the potential influence of implant design and manufacturer variability on DIB. Second, other components of the peri‐implant phenotype (Avila‐Ortiz et al. 2020), such as keratinized mucosal width or peri‐implant bone thickness, were not evaluated, which may have influenced the observed outcomes. Third, the retrospective nature of the study introduces inherent limitations, including the inability to control for all potential confounding factors, such as variations in prosthetic design (Katafuchi et al. 2018; Yi et al. 2020) or the exact number of alcoholic beverages consumed (Galindo‐Moreno et al. 2005) that could impact DIB. Fourth, future clinical studies should incorporate standardized and reproducible outcome assessment methods to further investigate the influence of additional factors on DIB (Tonetti et al. 2023). Finally, evaluation based solely on periapical radiographs may lead to inaccuracies. Therefore, the observed marginal bone level measurements (recorded in some cases 0.1 mm) and their association with marginal misfit should be interpreted with caution. These findings may not fully capture the true clinical accuracy and should be complemented by additional clinical parameters in routine practice to ensure a more comprehensive and reliable assessment (Dias et al. 2012; Walton 2023; Walton and Layton 2018).

## 5 Conclusions

Digital radiographic analysis indicates a strong association between the presence and size of vertical misfit gaps of ISPs and peri‐implant crestal bone levels. ISP without radiographic gaps exhibited lower DIB values, whereas gaps of ≥ 0.1 mm were significantly associated with greater DIB. A larger vertical gap increases the likelihood of peri‐implant crestal bone loss. These findings provide quantitative evidence of the biological consequences of accepting suboptimal prosthetic fit. Additionally, smoking and a history of periodontitis emerged as independent risk factors contributing to increased DIB.

## Author Contributions

**Emilio Couso‐Queiruga:** writing – original draft, validation, data curation, visualization, methodology. **Christoph A. Ramseier:** writing – review and editing, formal analysis, methodology, validation, visualization, conceptualization. **Vivianne Chappuis:** writing – review and editing, methodology, formal analysis. **Simone F. M. Janner M:** writing – review and editing, methodology, formal analysis. **Daniel Buser:** writing – review and editing, methodology. **Urs Brägger:** writing – review and editing, methodology. **Giovanni E. Salvi:** supervision, methodology, validation, visualization, writing – review and editing, conceptualization.

## Conflicts of Interest

The authors declare no conflicts of interest.

## Data Availability

The data that support the findings of this study are available on request from the corresponding author. The data are not publicly available due to privacy or ethical restrictions.
